# Parental Involvement, Teacher Public Praise, and Bullying Victimization Among Primary School Students: Evidence from China

**DOI:** 10.3390/bs16081267

**Published:** 2026-07-23

**Authors:** Long Diao, Zhaobiao Zong, Yanan Wang, Weihao Wang

**Affiliations:** 1 School of Politics and Public Administration, Guangxi Normal University, Guilin 541006, China; eminem7436520@gxnu.edu.cn; 2 Western Urban and Rural Integration Development Institute, Guangxi Normal University, Guilin 541006, China; 3 Guangxi Higher Education Development Research Center, Guangxi Normal University, Guilin 541006, China; 4 College of Education, Huaibei Normal University, Huaibei 235000, China; zzbpsy@chnu.edu.cn; 5 Faculty of Education, East China Normal University, Shanghai 200241, China

**Keywords:** parental involvement, school bullying victimization, primary school students, instrumental variables

## Abstract

Using cross-sectional survey data from 15,655 primary school students in China, this study examines whether more intensive parental involvement is associated with greater bullying victimization in school and whether teacher public praise statistically accounts for part of this association. Results from instrumental-variable, exploratory mediation, and moderation analyses suggest that higher parental involvement is linked to greater bullying victimization. Teacher public praise partially mediates this association, and the positive association between parental involvement and bullying victimization is stronger among students with higher emotional sensitivity.

## 1. Introduction

Bullying victimization refers to repeated physical, verbal, relational, or other forms of aggression in school contexts, typically involving a power imbalance that leaves victims unable to defend themselves effectively (Olweus, 1993). With the expansion of students’ online interactions, bullying can also occur through digital communication and spill over beyond school boundaries (Gladden et al., 2014). A large body of research has documented that bullying exposure is associated with a range of adverse developmental outcomes and may produce persistent harms that extend beyond the school years, which makes bullying prevention a continuing priority for education systems (Juvonen et al., 2011; Vassallo et al., 2014).

Parental involvement is widely regarded as a protective factor in bullying prevention because it can strengthen home–school communication and increase adults’ capacity to identify and respond to peer problems. Yet prior studies report only modest or inconsistent associations between parental involvement and bullying outcomes (Evans et al., 2014; Huang et al., 2019; Choi, 2024). This inconsistency suggests that the consequences of parental involvement may depend on how it is enacted and made visible within school settings.

There are two major reasons why existing evidence remains inconclusive. First, parental involvement, teacher public praise, and bullying victimization may be related in more than one temporal sequence. Bullying experiences may trigger parents’ increased monitoring and school contact, while parental actions may simultaneously reshape peer dynamics and students’ social positioning (Estevez et al., 2005; Lereya et al., 2013). Teachers may also publicly praise socially vulnerable students as a form of encouragement or protection, which makes reverse or reciprocal associations plausible. Second, possible explanatory processes are often underspecified. In particular, intensive parental involvement may increase teachers’ visible attention to the child, which may unintentionally make the child appear especially aligned with teacher attention and invite peer distancing or jealousy (Calarco, 2020; Klimecká, 2023). This risk may be further amplified among students who are more emotionally sensitive and therefore more likely to interpret social cues as stigmatizing and internalize negative labels (Galinsky et al., 2013).

To address these gaps, we use cross-sectional survey data from 15,655 primary school students in urban and rural China and apply instrumental-variable estimation together with exploratory mediation and moderation analyses. We examine whether more intensive parental involvement is associated with higher levels of bullying victimization after addressing one source of endogeneity concern, whether teacher public praise statistically accounts for part of this association, and whether emotional sensitivity conditions the strength of the association between parental involvement and bullying victimization. Because the data are cross-sectional, the analyses do not establish temporal precedence among parental involvement, teacher public praise, emotional sensitivity, and bullying victimization. The study therefore identifies theoretically informed associations rather than definitive causal processes.

### 1.1. Literature Review and Hypothesis Development

#### 1.1.1. Parental Involvement and Bullying Victimization: From Protective Resource to Potential Risk

Parental involvement has long been positioned as a protective resource in students’ school adjustment. Involvement can enhance monitoring, improve home–school communication, and increase adults’ capacity to detect and respond to peer problems, which may reduce bullying exposure (Hoover-Dempsey & Sandler, 1995; Jordan & Austin, 2012). Consistent with this view, meta-analytic and experimental evidence indicates that parent components can contribute to bullying prevention, although average associations tend to be modest and vary across programs and contexts (Huang et al., 2019; Van Niejenhuis et al., 2020).

Building on this mixed evidence, we shift attention from whether parental involvement is beneficial on average to the conditions under which it may carry unintended social costs. We argue that parental involvement may become counterproductive when it is intensive, highly visible, or strategically oriented toward securing advantages for a child.

This possibility is particularly plausible in school contexts where parental involvement is intertwined with status competition and close teacher alignment. Research on “helicopter” or privileged involvement emphasizes that socioeconomically advantaged parents may be more likely to maintain frequent contact with teachers, participate in school activities, and intervene in school processes to protect or promote their children’s interests (Calarco, 2020). Related work in China further suggests that “more” involvement can produce “less” favorable outcomes, consistent with a non-linear or threshold pattern in which excessive involvement carries unintended costs (Du & Zhang, 2025; A. Li et al., 2023). Prior research has shown that parental involvement is not uniformly beneficial and may, under some conditions, generate unintended social consequences for children in school settings. Building on this line of reasoning, we argue that more intensive parental involvement may be positively associated with bullying victimization.

**Hypothesis** **1.** *More intensive parental involvement will be positively associated with students’ bullying victimization.*

#### 1.1.2. Teacher Public Praise, Visibility, and Peer Interpretation

To explain why parental involvement may be positively associated with bullying victimization, we focus on teacher public praise as a possible school-based visibility process. This argument does not assume that public praise is inherently harmful. Teacher praise can encourage effort, reinforce classroom participation, support struggling students, and contribute to a positive classroom climate. Prior research also cautions that the meaning and consequences of praise depend on its content, timing, audience, and classroom context (Brophy, 1981; Henderlong & Lepper, 2002). Thus, public praise may have positive, negative, or context-dependent social consequences.

Our argument is that public praise may become socially risky under particular classroom conditions. First, from a labeling perspective, repeated public praise can make a student visibly associated with teacher attention. When this recognition is interpreted by peers as special treatment or as evidence of unusually close teacher–student alignment, the praised student may acquire a socially salient label. This interpretation is related to, but not identical with, the teacher’s pet and differential teacher treatment literature. Studies on perceived teacher favoritism suggest that students are sensitive to unequal teacher attention and may interpret selected students’ closeness to teachers as preferential treatment, especially when the favored student is not already accepted as a peer leader (Babad, 1995; Trusz, 2017).

Second, public praise may function as social-comparison information. Because praise is delivered in front of the whole class, it may signal not only approval from the teacher but also relative standing among classmates. Social comparison theory suggests that classrooms are important settings in which students evaluate themselves and others through visible cues of performance, recognition, and status (Dijkstra et al., 2008). In this sense, public praise may become meaningful to peers because it makes differences in teacher recognition observable.

Third, public praise may intersect with classroom status hierarchies and peer reputation. Research on peer aggression and status competition suggests that aggression can be used to maintain or contest social position, and that classroom hierarchy shapes how aggressive and victimized students are perceived (Ahn et al., 2010; Faris & Felmlee, 2011). Public teacher recognition may therefore generate admiration or acceptance when the praised student is already well liked or viewed as deserving, but it may generate distancing, resentment, or labeling when the praised student is perceived as undeserving, overly aligned with adults, or socially vulnerable.

Accordingly, we do not claim that peer jealousy, resentment, or teacher’s-pet labeling is directly observed in the present study. Rather, we use labeling theory, social comparison theory, and the literature on teacher favoritism and classroom status to provide competing theoretical interpretations of an observed statistical association. Public praise may reflect student effort, vulnerability, teacher support, or classroom participation; it may also make adult recognition visible in ways that reshape peer interpretation. We therefore formulate the mediation hypothesis in exploratory and associational terms.

**Hypothesis** **2.** *Teacher public praise in front of the whole class will statistically mediate the association between parental involvement and bullying victimization.*

#### 1.1.3. Emotional Sensitivity as a Moderator of the Association Between Parental Involvement and Bullying Victimization

Students vary in how they interpret and respond to adult involvement, social evaluation, and peer reactions. Emotional sensitivity is particularly relevant because students with higher sensitivity tend to perceive interpersonal cues more intensely and may respond more strongly to signs of rejection, stigma, or social differentiation. Prior research indicates that heightened sensitivity is associated with greater vulnerability in bullying contexts and with more adverse responses to negative peer experiences (Bondü et al., 2016; Jiang et al., 2022).

Intensive parental involvement may be especially consequential for emotionally sensitive students. Frequent parent–teacher contact, parental involvement in class affairs, and other visible forms of school-facing involvement may make students more attentive to how peers interpret their relationships with adults. Emotionally sensitive students may be more likely to perceive peer reactions to such involvement as stigmatizing, to internalize negative social labels, and to experience difficulties maintaining peer relationships (Galinsky et al., 2013; Theriot et al., 2005). Accordingly, the association between parental involvement and bullying victimization may be stronger among students with higher emotional sensitivity.

**Hypothesis** **3.** *Emotional sensitivity will moderate the association between parental involvement and bullying victimization.*

#### 1.1.4. Heterogeneity by Family Resource Conditions

Finally, the association between parental involvement and bullying victimization may vary across family contexts. Family socioeconomic resources are related to children’s exposure to bullying, although these associations are generally modest and vary across bullying roles (Tippett & Wolke, 2014). Family resources also shape parents’ capacity to communicate with teachers, participate in school activities, and intervene in school processes, suggesting that similarly intensive involvement may carry different meanings across social contexts (Calarco, 2020; A. Li et al., 2023).

Family structure and hukou status are particularly relevant in China. Research on resource dilution shows that the arrival of a sibling can reduce the resources and investments available to each child, implying that only children may receive more concentrated family investment (Chen, 2020). Urban–rural differences in students’ educational experiences are also associated with differences in sibling structure, parental education, and parent–teacher interaction (Zhao et al., 2017). These conditions may be associated with both the intensity of school-facing parental involvement and its visibility to teachers and peers. We therefore examine whether the association between parental involvement and bullying victimization differs by only-child status, hukou, socioeconomic status, cultural capital, and migrant status. Because the existing literature does not provide a consistent basis for predicting the direction of each difference, we formulate a general heterogeneity hypothesis:

**Hypothesis** **4.** *The association between parental involvement and bullying victimization will vary across family structure, hukou status, and family-resource conditions.*

**Figure 1:**
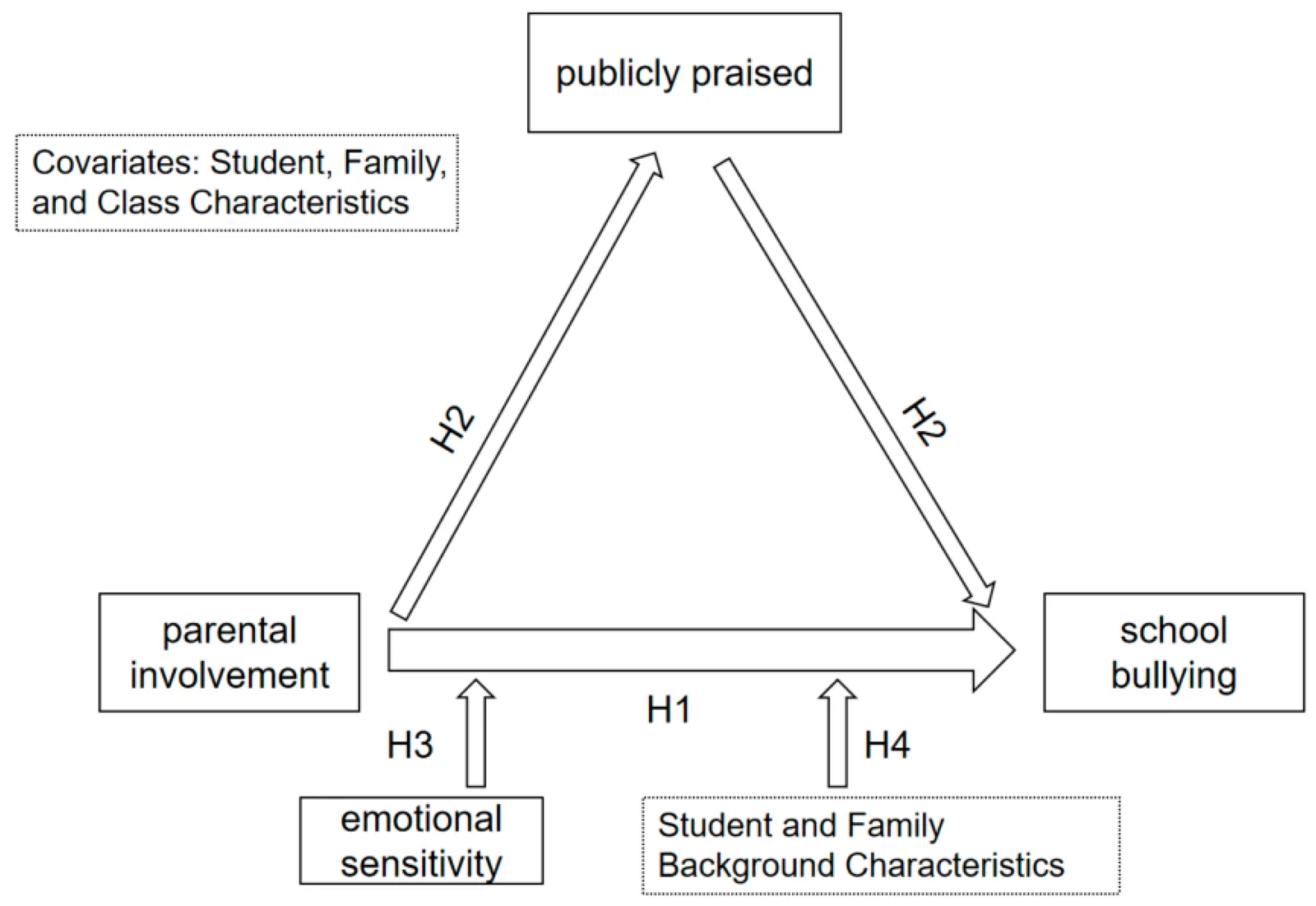
Hypothesized associational model of parental involvement, teacher public praise, emotional sensitivity, and bullying victimization.

Based on this framework, Figure 1 presents the hypothesized model. H1 denotes the direct association between parental involvement and bullying victimization. H2 denotes the mediating indirect association through teacher public praise. H3 denotes the moderating association of emotional sensitivity on the association between parental involvement and bullying victimization. H4 denotes heterogeneity in the association between parental involvement and bullying victimization across student and family background characteristics. Student-, family-, and class-level covariates are included in all models.

## 2. Methods

### 2.1. Sample and Data Collection

We use data from the Reading and Primary School Student Development Project conducted in Hefei, Anhui Province, China. In 2020, the per capita GDP of Anhui Province was 63,426 yuan (approximately 9195 USD), ranking 13th among the 31 mainland provinces (CNBS, 2021). Primary school students in Anhui accounted for 4.4% of the national total. Student surveys were administered in two waves, with urban schools surveyed in December 2020 and rural schools in March 2021. Student surveys were administered in two waves: urban schools were surveyed in December 2020 and rural schools in March 2021. Because survey timing is linked to the urban–rural sampling sequence, school location and survey timing cannot be fully separated. Both waves were conducted within a relatively short interval during the COVID-19 pandemic, and fieldwork records did not indicate major differences in school routines or survey administration procedures across the two periods. Nevertheless, urban–rural and hukou-related subgroup findings are interpreted cautiously. The final analytic sample includes 15,655 students in Grades 4–6 from 45 primary schools and 358 classes.

The study uses cross-sectional observational survey data. Accordingly, the analyses estimate associations among parental involvement, teacher public praise, emotional sensitivity, and bullying victimization at one point in time. Although the instrumental-variable specification addresses one source of endogeneity concern, the design does not establish temporal precedence. The mediation and moderation analyses are therefore interpreted as exploratory tests of theoretically informed associations rather than as evidence of causal mechanisms.

### 2.2. Measurements

The variables used in this study are presented in Table 1. The dependent variable is school bullying, operationalized as students’ self-reported bullying-related victimization. The measure includes eight items asking whether classmates had engaged in the following behaviors toward the student: teasing or verbal abuse, exclusion from play, spreading rumors, stealing belongings, physical harm such as kicking, hitting, or pushing, forcing the student to do something unwillingly, spreading embarrassing information, and threatening the student. The eight items were then summed to create an overall index ranging from 0 to 24.

The items cover verbal, relational, property-related, physical, coercive, and threatening forms of peer victimization. Internal consistency was acceptable, with Cronbach’s alpha = 0.87. Factor-analytic results also supported the use of an overall index. The full item wording and coding scheme are reported in Supplementary Table S2. Because the items measure students’ reported exposure to bullying-related behaviors but do not directly assess perceived power imbalance, the measure should be interpreted as a frequency-based index of school bullying victimization or bullying-related peer victimization rather than a complete behavioral diagnosis of bullying in the strict definitional sense. We therefore use the term “school bullying” as shorthand for students’ self-reported bullying-related victimization throughout the empirical analysis.

Table 1 provides an overview of the sample profile and the distributions of the main variables. The students had a mean age of 11.41 years, and 54.1% were male. Urban-hukou students accounted for 53.8% of the sample, while 66.6% held local hukou. Only children represented 62.9% of the sample, and 56.3% of parents were employed full time. Parental involvement was relatively common: 70.9% of students had at least one reported form of school-facing parental involvement, and the mean involvement-intensity score was 1.25 on a scale from 0 to 3. Mothers were the primary learning supervisors in 64.4% of households. The mean bullying-victimization score was 6.61, while the mean frequency of teacher public praise was 2.46. The substantial variation in parental involvement, bullying victimization, and the focal family-background characteristics provides a useful basis for the subsequent regression and heterogeneity analyses. At the same time, the sample remains geographically bounded to the study area and should not be interpreted as nationally representative.

The key independent variable, parental involvement, is operationalized as school-facing parental involvement. It is an intensity index based on three indicators: (a) whether parents proactively contact teachers, (b) whether teachers proactively contact parents, and (c) whether parents serve as members of the class committee. Responses were coded and summed so that higher values represent more intensive involvement. For robustness checks, we also recode parental involvement as a binary indicator reflecting whether the family shows any involvement in these domains.

To address potential endogeneity in the association between parental involvement and bullying victimization, we use “*whether the mother is the primary person responsible for supervising the student’s learning*” as an instrumental variable. We also include “*public praise in front of the whole class*” as a mediating variable and “*emotional sensitivity*” as a moderating variable to further examine possible explanatory processes linking parental involvement and bullying victimization. Drawing on labeling theory, we posit that students who receive public praise in front of the class are more likely to become targets of bullying victimization. Furthermore, based on prior findings, emotionally sensitive students are more susceptible to self-labeling associations, which is associated with higher levels of their likelihood of victimization (X. Wang et al., 2023; Zhang et al., 2026).

Emotional sensitivity was measured using ten student-reported items from the Reading and Primary School Student Development Project questionnaire. These items capture students’ tendency to perceive, understand, and be affected by others’ emotions, as well as their emotional reactivity to affective scenes or peer emotions. The items include statements such as feeling sad after spending time with a friend who is feeling down, being easily affected by others’ emotions, noticing when friends feel afraid or angry, and feeling sad when watching sad scenes on television or in movies. The full item wording, response options, and recoding scheme are reported in Supplementary Table S2. The ten recoded items were then averaged to create a continuous emotional-sensitivity score ranging from 1 to 5. We use the continuous measure in the main moderation analysis because no validated threshold is available for dichotomizing the scale. The measure showed acceptable internal consistency, with Cronbach’s alpha = 0.91. As a sensitivity check, we also re-estimated the moderation model using the dichotomized measure used in the original specification; these results are reported in Supplementary Table S5.

Finally, we include a set of covariates at the student, family, and class levels. This choice is guided by prior research showing that bullying victimization is shaped by individual characteristics, family conditions, and school context. We also included student-, family-, and class-level covariates shown in Table 1. Student-level covariates include gender, age, and previous-semester academic performance (Juvonen et al., 2011; Tippett & Wolke, 2014). Family-level covariates include parent age, parental education, employment, hukou status, household income, book collection, parent occupation type, only-child status, household size, and the proportion of school-age children (Lereya et al., 2013; Tippett & Wolke, 2014; Yang et al., 2019). Class-level covariates include homeroom teacher gender, teaching experience, professional title, and class size (Estevez et al., 2005; Y. J. Wang & Chen, 2023). These covariates were included in the multivariate models to account for observed background differences associated with parental involvement and bullying victimization.

### 2.3. Analytic Strategies

#### 2.3.1. Accounting for the Hierarchical Data Structure

Because the data have a hierarchical structure, with students nested within classes and schools, observations from the same classroom or school may not be statistically independent. Bullying victimization, teacher public praise, and parental involvement may be shaped by shared classroom climate, teacher practices, peer composition, and school context. We therefore first estimated unconditional random-intercept models to calculate intraclass correlation coefficients (ICCs) for the main variables. The ICCs were calculated for school bullying, parental involvement, teacher public praise, and emotional sensitivity to assess the extent to which these variables varied across classes and schools.

To account for within-school dependence in the regression analyses, all main models were re-estimated using school-level cluster-robust standard errors. We selected school-level clustering because classes are nested within schools, and school-level clustering allows arbitrary correlation among students within the same school, including correlation arising from shared classroom and school environments. This approach preserves the comparability of the OLS, instrumental-variable, mediation, moderation, heterogeneity, and robustness models while correcting the statistical inference for the nested data structure. The clustering level is indicated in the notes to all regression tables.

#### 2.3.2. Ordinary Least Squares Regression

The empirical analysis proceeds in four stages and reports 11 main models. First, Models (1) and (2) estimate the baseline association between parental involvement and bullying victimization using ordinary least squares regression, without and with the full set of covariates, respectively. Second, Models (3) and (4) implement the instrumental-variable analysis: Model (3) reports the first-stage association between the instrument and parental involvement, while Model (4) reports the second-stage association between instrumented parental involvement and bullying victimization. Third, Models (5)–(9) examine the proposed mediation and moderation analyses. Models (5)–(7) test the mediating role of teacher public praise, Model (8) tests whether emotional sensitivity moderates the direct association between parental involvement and bullying victimization, and Model (9) tests whether emotional sensitivity moderates the association between parental involvement and bullying victimization. Fourth, Models (10) and (11) use the binary measure of parental involvement to report heterogeneity by only-child status and urban hukou, the two dimensions for which significant or marginally significant interaction associations were detected. In addition, Models (12) and (13), reported in Supplementary Table S1, assess the robustness of the baseline results using a binary measure of parental involvement.

First, Models (1) and (2) estimate the baseline association between parental involvement and bullying victimization using ordinary least squares (OLS). Model (1) presents the unadjusted specification, while Model (2) includes the full set of student-, family-, and class-level covariates. The model is specified as follows (Equation (1)):

(1) $${Bully}_{i}=\alpha +\beta_{1}{FSC}_{i}+\beta_{2}X_{i}+\varepsilon_{i}$$

As shown in Equation (1), *Bully_i_* represents the level of student *i*-reported school bullying victimization, while *FSC_i_* denotes the degree of parental involvement for student *i*. *X_i_* represents the vector of student-level, family-level, and class-level covariates listed in Table 1 and described in Section 2.2, and *ε_i_* is the random error term.

#### 2.3.3. Instrumental Variable Regression

Second, Models (3) and (4) address the potential endogeneity of parental involvement using two-stage least squares estimation. Model (3) reports the first-stage equation, and Model (4) reports the second-stage outcome equation. The fundamental idea behind instrumental variable (IV) regression is to identify an instrument that satisfies both relevance and exogeneity. In other words, the instrument should be strongly correlated with the endogenous independent variable but uncorrelated with the error term; ideally, it is associated with the dependent variable only through its association with the endogenous variable rather than through a direct association (Angrist & Krueger, 1991).

To address potential endogeneity in the association between parental involvement and bullying victimization, we use “*whether the mother is the primary person responsible for supervising the student’s learning*” as an instrumental variable. The relevance condition is theoretically plausible because, in the Chinese family context, mothers often assume primary responsibility for children’s educational supervision and daily academic monitoring (Offer & Schneider, 2011; X. Li, 2020). Mothers may also be more willing to invest time and resources in children’s schooling (Dizon-Ross & Jayachandran, 2023). When the mother is the main learning supervisor, the family is therefore more likely to maintain school-facing contact with teachers and participate in class-related affairs. In addition, school personnel may be more likely to contact mothers when children experience academic or social problems at school, which further links maternal supervisory responsibility to parental involvement in school life (Buzard et al., 2025).

**Figure 2:**
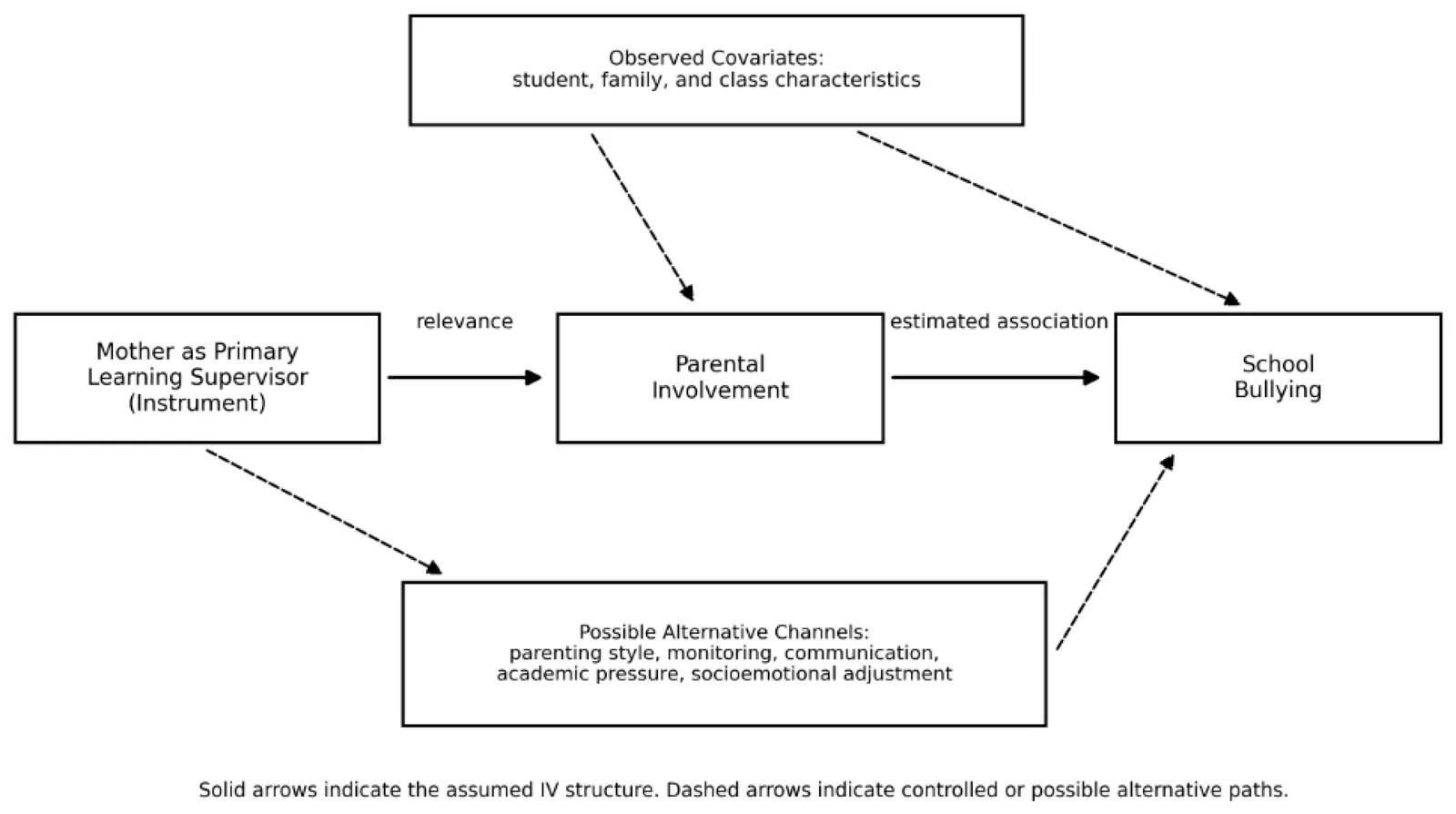
Assumed identifying structure for the instrumental-variable analysis.

The exclusion restriction is more demanding and cannot be directly tested. Our identifying assumption is that, conditional on the observed student-, family-, and class-level covariates, maternal responsibility for supervising learning is associated with bullying victimization only through school-facing parental involvement. Figure 2 summarizes this assumed identifying structure. The solid arrows show the intended IV logic: maternal responsibility for supervising learning is expected to be associated with parental involvement, and parental involvement is then associated with bullying victimization. The dashed arrows indicate possible alternative channels that could threaten the exclusion restriction, such as parenting style, monitoring intensity, parent–child communication, academic pressure, and children’s socioemotional adjustment. Thus, the figure is intended not to prove the validity of the instrument but to make the identifying assumption and its potential limitations explicit.

Nevertheless, plausible violations of the exclusion restriction remain. Maternal supervision may be associated with parenting style, monitoring intensity, parent–child communication, academic pressure, family conflict, attachment, or children’s socioemotional adjustment, each of which may also be related to bullying victimization. For this reason, the IV results should be interpreted cautiously as endogeneity-aware associational estimates rather than definitive causal effects. We therefore report additional first-stage diagnostics and interpret the IV estimates cautiously, while acknowledging that these diagnostics cannot verify the exclusion restriction. The two-stage least squares (2SLS) estimation process is specified in Equations (2) and (3).

(2) $${FSC}_{i}=\pi_{0}+\pi_{1}{IV}_{i}+\pi_{2}X_{i}+\nu_{i}$$

(3) $${\mathrm{Bully}}_{i}=\alpha +\beta_{1}{\hat{FSC}}_{i}+\beta_{2}X_{i}+\varepsilon_{i}$$

Equation (2) uses “*whether the mother is the primary person responsible for supervising the student’s learning*” (*IV_i_*) as the instrumental variable to predict the level of parental involvement (*FSC_i_*). Equation (3) then uses the predicted parental involvement from the first stage ($\hat{FSC}$*_i_*) to estimate the level of bullying victimization experienced by student *i* (*Bully_i_*). Instrument relevance is assessed using the first-stage F statistic. Following the conventional rule of thumb, an F statistic above 10 is treated as evidence that weak identification is unlikely. We also conduct the Durbin–Wu–Hausman test to examine whether parental involvement should be treated as endogenous. A statistically significant test rejects the null hypothesis of exogeneity and supports the use of the instrumental-variable specification rather than OLS alone. The first-stage F statistic, partial R^2^, and Durbin–Wu–Hausman test are used to assess instrument relevance and the need for an instrumental-variable specification, but they do not verify the exclusion restriction. The exclusion restriction remains an identifying assumption rather than an empirically testable condition in the present exactly identified model. We therefore interpret the IV estimates cautiously as endogeneity-aware associational evidence rather than definitive causal evidence.

#### 2.3.4. Mediation and Moderation Analyses

Models (5)–(7) examine the exploratory statistical mediation pattern involving teacher public praise. Model (5) estimates the association between parental involvement and teacher public praise, Model (6) estimates the association between teacher public praise and bullying victimization, and Model (7) includes both parental involvement and teacher public praise in the outcome equation. The indirect association is calculated as the product of the two pathway coefficients and assessed using a nonparametric bootstrap procedure with 5000 resamples. The Sobel test is retained only as a supplementary check. Because the data are cross-sectional and the mediation models do not fully incorporate the instrumental-variable identification strategy, these results are interpreted as exploratory statistical mediation rather than causal mediation. The full decomposition of the total, direct, and indirect associations is reported in Supplementary Table S6.

Models (8) and (9) test the moderating role of emotional sensitivity. Emotional sensitivity is treated as a continuous measure in the main analysis, and parental involvement and emotional sensitivity are mean-centered before constructing the interaction term. A positive interaction coefficient indicates that the association between parental involvement and bullying victimization is stronger among students with higher emotional sensitivity. We additionally report a sensitivity analysis using the dichotomized emotional-sensitivity measure in Supplementary Table S5.

Equation (4) specifies the mediator equation, in which parental involvement predicts teacher public praise. Equation (5) specifies the mediation outcome equation, in which parental involvement and teacher public praise jointly predict bullying victimization. Equation (6) specifies the moderation model, in which bullying victimization is regressed on parental involvement, emotional sensitivity, and their interaction. All models include the student-, family-, and class-level covariates described in Section 2.2.

(4) $$M_{i}=\alpha +\beta_{1}{FSC}_{i}+\beta_{2}X_{i}+\varepsilon_{i}$$

(5) $${Bully}_{i}=\alpha +\beta_{1}{FSC}_{i}+\beta_{2}M_{i}+\beta_{3}X_{i}+\varepsilon_{i}$$

(6) $${Bully}_{i}=\alpha +\beta_{1}{FSC}_{i}+\beta_{2}m_{i}+\beta_{3}{FSC}_{i}*m_{i}+\beta_{4}X_{i}+\varepsilon_{i}$$

#### 2.3.5. Heterogeneity Analysis

Fourth, we examine whether the association between parental involvement and bullying victimization varies across student and family background characteristics. For ease of interpretation, the heterogeneity analyses use the binary measure of parental involvement, coded as 0 for no school involvement and 1 for any school involvement. Separate interaction models are estimated for only-child status, urban hukou, local versus non-local hukou status, annual per capita household income, and household book collection size. Each model includes parental involvement, the relevant background characteristic, their interaction term, and the full set of covariates. Models (10) and (11) report the results for only-child status and urban hukou, respectively.

(7) $${Bully}_{i}=\alpha +\beta_{1}{FSC}_{i}+\beta_{2}({FSC}_{i}*X_{i})+\beta_{3}X_{i}+\varepsilon_{i}$$

#### 2.3.6. Robustness Test

Finally, we examine whether the baseline results are sensitive to the operationalization of parental involvement. We recode parental involvement as a binary indicator that distinguishes students whose parents report any form of school involvement from those whose parents report no involvement. Models (12) and (13) then re-estimate the unadjusted and fully adjusted OLS specifications using this alternative measure. Consistency in the direction and statistical significance of the estimates is treated as evidence that the main findings are not driven by the original intensity-based coding of parental involvement.

Because the dependent variable is a bounded nonnegative score ranging from 0 to 24, we examined its empirical distribution before estimating the regression models. We report the mean, standard deviation, skewness, kurtosis, and proportion of zero scores for the school bullying measure. We also inspected OLS residual diagnostics, including residual-versus-fitted plots and the distribution of residuals.

OLS is retained as the primary specification because the outcome is a summed victimization index rather than a single event count, and OLS coefficients provide a direct interpretation in terms of differences in the school bullying score. In addition, the large sample size and the use of school-level cluster-robust standard errors help reduce concerns about statistical inference under nonnormal residuals. Nevertheless, because the outcome is bounded, nonnegative, and potentially overdispersed, we conduct robustness checks using negative binomial regression. These models assess whether the substantive conclusions are sensitive to using a generalized linear model that is more appropriate for skewed count-like outcomes.

We examined the extent of missingness for the analytic variables and found that missingness was minimal, with variable-level missing rates ranging from 0.5% to 1%. Given the low level of missing data, we used complete-case analysis based on listwise deletion for the regression models. We also compared the complete-case estimates with the earlier specifications and found no substantive changes in the direction or interpretation of the main results.

To assess potential multicollinearity, we calculated variance inflation factors (VIFs) for the fully adjusted regression model including the principal variables and the full set of covariates. For models involving interaction terms, the relevant continuous variables were mean-centered before constructing the interaction terms. All VIF values were below the conventional threshold of 10, with a maximum VIF of 3.68 and a mean VIF of 1.61, suggesting that multicollinearity is unlikely to drive the main estimates.

## 3. Results

The amount of missing data was minimal across the analytic variables, and complete-case estimates were substantively consistent with the original specifications. The results reported below are therefore based on complete-case analyses without mean imputation. Before estimating the regression models, we assessed the hierarchical structure of the data using unconditional random-intercept models. The school-level ICCs were 0.05 for school bullying, 0.03 for parental involvement, 0.05 for teacher public praise, and less than 0.01 for emotional sensitivity. The corresponding class-level ICCs were 0.08, 0.05, 0.14, and 0.03, respectively. These results indicate that the main variables show nonzero clustering at the classroom and school levels, supporting the use of school-level cluster-robust standard errors in the regression analyses. We also examined the distribution of the school bullying score before estimating the regression models. The score ranged from 0 to 24, with a mean of 6.61 and a standard deviation of 6.69. The skewness was 0.80, the kurtosis was 2.90, and only 2% of students had a score of zero, suggesting that zero inflation is not severe. These diagnostics indicate that the outcome is bounded and somewhat skewed, supporting the need for robustness checks beyond OLS. Residual diagnostics for the OLS models also showed some departure from normality, which is expected for a bounded victimization score. We therefore retain OLS as the main specification for interpretability and comparability, while reporting negative binomial robustness checks in Supplementary Table S7. The negative binomial results are substantively consistent with the OLS estimates, indicating that the main conclusions are not driven by the linear-model specification.

### 3.1. Association Between Parental Involvement and Bullying Victimization Among Primary School Students

All regression tables report school-level cluster-robust standard errors. Models (1) and (2) present the ordinary least squares regression results for the association between parental involvement and bullying victimization among primary school students, with and without the inclusion of control variables. As shown in Table 2, higher levels of parental involvement are associated with an increased likelihood of students experiencing bullying victimization. This association contrasts with the conventional expectation that parental involvement is uniformly protective against bullying victimization, underscoring the need for cautious endogeneity-aware estimation and further indirect association analysis. All regression models reported below use school-level cluster-robust standard errors to account for the nested structure of the data.

### 3.2. Results of Instrumental Variable Regression

Table 3 presents the instrumental-variable regression results. The first-stage coefficient for maternal responsibility for supervising learning is 0.239, indicating that students whose mothers are the primary learning supervisors report higher levels of school-facing parental involvement. The first-stage F statistic is 202.31, and the partial R^2^ is 0.013, suggesting that the instrument is strongly associated with parental involvement. Because the model is exactly identified, an overidentification test is not applicable.

In Model (4), the IV estimate for parental involvement is positive and statistically significant. This pattern is consistent with Hypothesis 1 and indicates that the positive association between parental involvement and bullying victimization remains in a specification that addresses one source of endogeneity concern. However, the exclusion restriction cannot be directly verified, and the diagnostics reported above do not establish that the instrument affects bullying victimization only through parental involvement. The IV result should therefore be interpreted cautiously as endogeneity-aware associational evidence rather than definitive causal evidence.

### 3.3. Results of the Mediation and Moderation Analyses

Table 4 reports the results of the mediation and moderation analyses. For the mediation analysis, the indirect association was evaluated using a nonparametric bootstrap procedure with 5000 resamples, while the Sobel test was retained only as a supplementary check. Model (5) shows that parental involvement is positively associated with more frequent public praise by teachers. Model (6) shows that public praise is positively associated with school bullying. In Model (7), parental involvement and public praise are entered simultaneously. Public praise remains positively associated with school bullying, while the coefficient for parental involvement is reduced from 0.222 in Model (2) to 0.208 but remains statistically significant.

The effect decomposition is reported in Supplementary Table S6. The estimated indirect association through teacher public praise is 0.014, with a bootstrap standard error of 0.004 and a 95% bootstrap confidence interval of [0.006, 0.023]. This indirect association accounts for approximately 6.4% of the total association. These results indicate a partial statistical mediation pattern: teacher public praise statistically accounts for a small but significant part of the association between parental involvement and school bullying, while most of the association remains direct or unexplained by this mediator. Because the mediation model is based on cross-sectional observational data and does not fully incorporate the instrumental-variable identification strategy, this result should be interpreted as exploratory statistical mediation rather than evidence of a causal mediation process. These findings support Hypothesis 2 and suggest that teacher-mediated public visibility constitutes one plausible indirect association linking parental involvement to students’ peer experiences.

Models (8) and (9) examine the moderating role of emotional sensitivity using the continuous measure. Model (8) includes the main effects of parental involvement and emotional sensitivity. Emotional sensitivity is positively associated with school bullying, indicating that students with higher emotional sensitivity report more frequent bullying-related victimization. Model (9) further introduces the interaction between parental involvement and emotional sensitivity. The interaction coefficient is positive and statistically significant, indicating that the positive association between parental involvement and school bullying is stronger among students with higher emotional sensitivity. This finding supports Hypothesis 3 and suggests that emotional sensitivity operates as a continuous moderator rather than merely reflecting an artificial group distinction. Supplementary Table S5 reports the sensitivity analysis using the dichotomized emotional-sensitivity measure. The interaction between parental involvement and the dichotomized emotional-sensitivity measure remains positive and statistically significant, which is consistent with the main continuous specification. Because no validated threshold exists for dichotomizing this measure, we treat the continuous specification as the preferred main analysis and use the dichotomized specification only as a supplementary sensitivity check.

### 3.4. Results of the Heterogeneity Analysis

As shown in Table 1, only children accounted for 62.9% of the sample and students with siblings for 37.1%, while urban- and rural-hukou students accounted for 53.8% and 46.2%, respectively. Both focal comparisons therefore include substantial numbers of students in each subgroup. Because multiple interaction terms were examined, we treat the heterogeneity analyses as exploratory and report unadjusted *p*-values.

Table 5 presents the heterogeneity results using the binary measure of parental involvement. Model (10) shows a positive interaction between parental involvement and only-child status. The Wald test for the interaction term was statistically significant, F(1, 44) = 4.60, *p* = 0.04. Supplementary Table S8 reports the corresponding marginal effects and average adjusted predicted values. The association between parental involvement and school bullying was negative but not statistically significant among students with siblings (b = −0.363, SE = 0.231, 95% CI [−0.829, 0.103], *p* = 0.123), whereas it was positive and statistically significant among only children (b = 0.255, SE = 0.119, 95% CI [0.015, 0.495], *p* = 0.038). The predicted values show that among only children, students with parental involvement had a higher predicted school bullying score than those without parental involvement.

Model (11) shows a positive but more tentative interaction between parental involvement and urban hukou. The Wald test for the interaction term was marginally significant, F(1, 44) = 3.33, *p* = 0.07. Supplementary Table S8 shows that the association between parental involvement and school bullying was negative and not statistically significant among rural-hukou students (b = −0.210, SE = 0.211, 95% CI [−0.636, 0.216], *p* = 0.326), but positive and statistically significant among urban-hukou students (b = 0.308, SE = 0.152, 95% CI [0.002, 0.614], *p* = 0.049). However, because the interaction term itself was only marginally significant, the urban-hukou heterogeneity result should be interpreted more cautiously than the only-child result. Additional interaction models for local hukou, annual per capita household income, and household book collection size yielded no statistically significant interaction associations.

Taken together, the exploratory heterogeneity evidence is concentrated mainly in only-child status and, more tentatively, urban hukou. These results are consistent with Hypothesis 4 in an exploratory sense but should not be interpreted as confirmatory evidence after multiple testing. One plausible interpretation is that only children and some urban-hukou students may receive more concentrated parental attention and school-oriented investment, which may heighten their social visibility and reduce opportunities for autonomous peer interaction. However, because urban–rural school location is partly confounded with survey timing and because hukou status does not fully capture school location or family resources, this interpretation should be treated as suggestive rather than definitive.

### 3.5. Robustness Checks

Supplementary Table S1 reports the robustness analysis using a binary operationalization of parental involvement. Parental involvement remains positively associated with bullying victimization in both the unadjusted and fully adjusted specifications, suggesting that the main finding is not driven by the original intensity-based coding of parental involvement. We further examined whether the association between parental involvement and school bullying was driven by a specific component of the formative index. Supplementary Table S3 reports the distribution and pairwise correlations of the three components: parent-initiated teacher contact, teacher-initiated parent contact, and class parent committee membership. The three components were positively but only moderately correlated, supporting our treatment of the index as formative rather than reflective. Supplementary Table S4 reports component-level regressions. When entered separately, all three components were positively associated with school bullying, but the association was most pronounced for teacher-initiated parent contact. When the three components were entered simultaneously, teacher-initiated parent contact remained the strongest and most stable predictor, while the coefficient for parent-initiated contact was substantially attenuated. This pattern suggests that the main association is not simply an association with proactive parental engagement. Rather, it should be interpreted as relating to school-facing forms of parental involvement, including reactive school contact that may occur when students experience academic, behavioral, or social difficulties.

**Figure 3:**
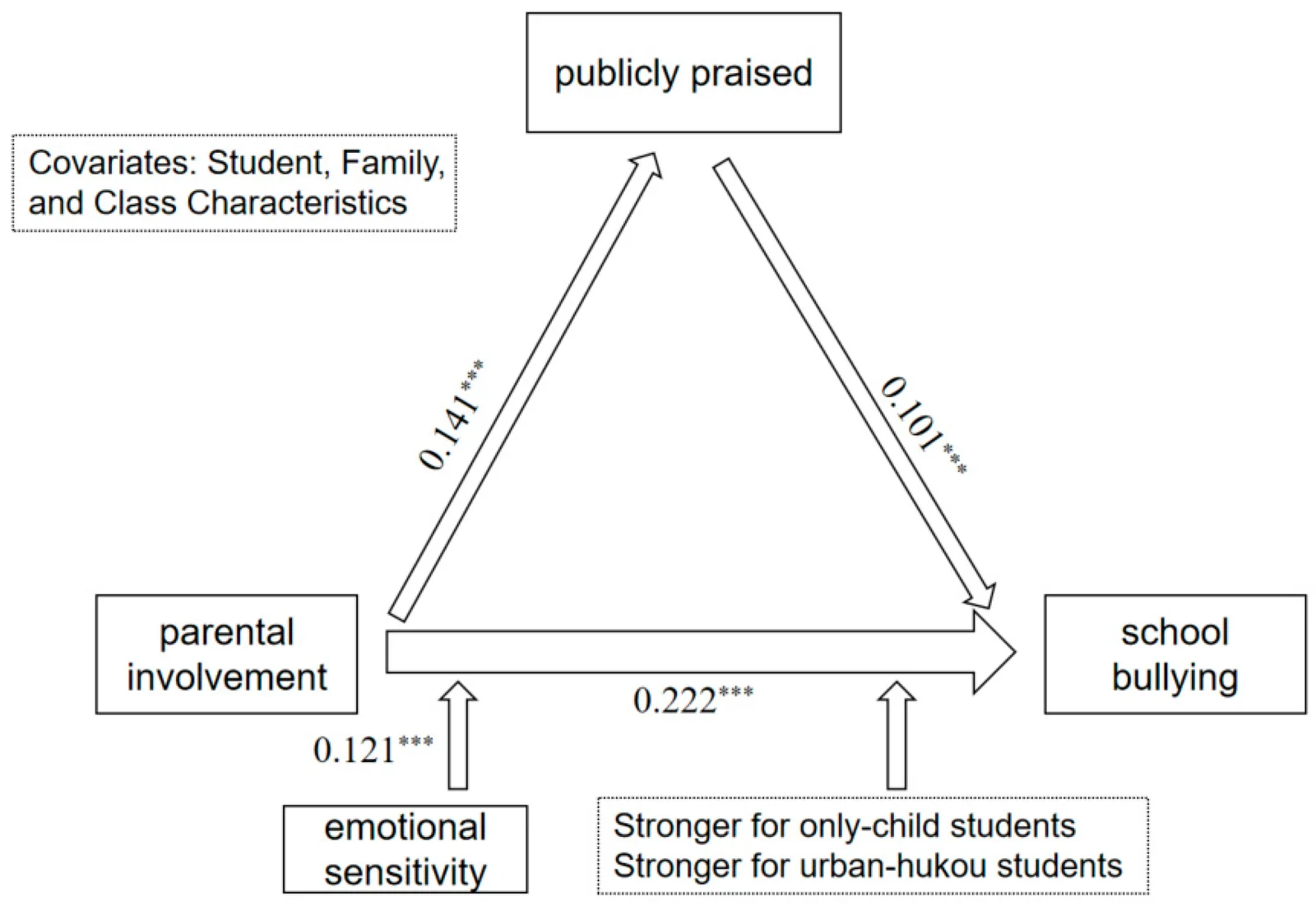
Empirical summary of the observed associations in the final models. *** *p* < 0.01.

Figure 3 summarizes the empirically supported paths and key coefficients from the final models, thereby providing an integrated view of the main results before turning to the discussion.

## 4. Discussion

Contrary to the common assumption that parental involvement is uniformly protective, this study finds that more intensive parental involvement is positively associated with bullying victimization among primary school students. This pattern remains in the instrumental-variable specification, although the IV estimate should be interpreted cautiously because the exclusion restriction cannot be empirically verified. The finding suggests that parental involvement may not operate as a uniformly beneficial resource in peer contexts, especially when it becomes highly visible in school interactions (Calarco, 2020; Du & Zhang, 2025; A. Li et al., 2023).

The mediation and moderation analyses provide exploratory evidence about possible explanatory processes. Teacher public praise statistically accounts for a small but significant part of the association between parental involvement and bullying victimization. One theoretically informed interpretation is that public recognition may make some students more visible to peers and may, under certain classroom conditions, invite social comparison, perceived favoritism, or unfavorable labeling (Klimecká, 2023; Galinsky et al., 2013). However, the study does not directly measure classmates’ motives, teachers’ reasons for giving praise, or the content and fairness of praise. Public praise may also reflect student effort, classroom participation, or teachers’ attempts to support vulnerable students. The moderation results further suggest that the positive association between parental involvement and bullying victimization is stronger among students with higher emotional sensitivity, who may be more attentive to social evaluation and peer reactions (Bondü et al., 2016; Jiang et al., 2022; Galinsky et al., 2013; Theriot et al., 2005).

Taken together, the findings across H1–H4 provide a layered account of how parental involvement may become associated with bullying victimization. H1 establishes the counterintuitive positive association between more intensive parental involvement and bullying victimization, while H2 identifies teacher public praise as one partial indirect association through which school-facing parental involvement may become visible within the peer environment. H3 further shows that this association is stronger among students with higher emotional sensitivity, indicating that the social consequences of parental involvement depend partly on how students perceive and respond to interpersonal cues. H4 receives partial support: the exploratory heterogeneity analyses suggest that the association is concentrated mainly among only children and, more tentatively, among urban-hukou students. These patterns are consistent with the idea that concentrated parental attention and school-oriented investment may heighten students’ social visibility, but they should not be interpreted as confirmatory evidence after multiple testing (Chen, 2020; Zhao et al., 2017). Overall, the findings point to a context-dependent process in which family–school involvement, visible teacher recognition, student sensitivity, and family background jointly shape children’s peer experiences.

### 4.1. Implications for Educational Practice

The practical implications should be interpreted cautiously. The findings do not imply that parental involvement should be discouraged or that teachers should reduce public praise. Rather, they suggest that schools should attend to how family–school involvement and teacher recognition are enacted within classroom peer ecologies. Public praise can be pedagogically valuable when it recognizes effort, encourages participation, and supports students’ confidence, but teachers may need to consider whether recognition is distributed equitably and whether repeated public attention makes particular students socially distinctive (Y. J. Wang & Chen, 2023).

Schools may therefore support more context-sensitive and non-stigmatizing recognition practices. Teachers can emphasize effort, improvement, cooperation, and diverse forms of contribution, while also cultivating classroom norms that discourage peer comparison, status labeling, and exclusion. These recommendations are tentative and hypothesis-generating. Longitudinal and experimental studies are needed before stronger practice guidelines can be offered regarding how specific forms of public praise affect peer relationships and bullying victimization.

### 4.2. Limitations and Future Directions

Several limitations should be noted. First, the data are cross-sectional and geographically bounded to one province, which limits temporal inference and generalizability. Although the theoretical model treats parental involvement as preceding teacher public praise and bullying victimization, reverse or reciprocal sequences remain possible. Bullying experiences may lead parents to contact teachers more frequently, and teachers may publicly praise students who are already socially or academically vulnerable.

Second, the instrumental-variable analysis relies on an exclusion restriction that cannot be empirically verified. Although maternal responsibility for learning supervision is strongly associated with parental involvement, it may also be related to parenting style, monitoring, parent–child communication, academic pressure, or students’ socioemotional adjustment. The IV estimates should therefore be interpreted as sensitivity evidence addressing one source of endogeneity concern rather than as conclusive causal evidence.

Third, the measures have limitations. Parental involvement captures school-facing involvement rather than the full range of home-based support or parenting practices. Bullying victimization is measured as a summed frequency index and does not directly assess perceived power imbalance or distinguish bullying types. Public praise is measured by frequency only and does not capture its content, fairness, distribution, or classroom context. Future research should use longitudinal and multisite designs, richer multidimensional measures, teacher and peer reports, and experimental or quasi-experimental designs to test the proposed processes more directly.

Overall, this study suggests that the consequences of parental involvement depend not only on how much parents participate in school life but also on how that involvement is translated into visible classroom practices and experienced by different students. The findings do not imply that schools should discourage parental involvement or teacher praise. Instead, they point to the value of family–school collaboration that is supportive without making students socially distinctive, recognition practices that are attentive to peer dynamics, and additional support for students who are particularly sensitive to social evaluation. Given the observational and geographically bounded nature of the data, these conclusions should be interpreted as evidence of a plausible and context-dependent process that requires further longitudinal and multisite testing.

## Figures and Tables

**Table 1:** Variable Description.

Variable	Mean	SD	Min	Max	Definition
Dependent Variable					
School Bullying	6.609	6.690	0	24	Eight-item summed index; possible scores range from 0 to 24, with higher scores indicating more frequent or severe bullying victimization
Independent Variable					
Parental Involvement (continuous)	1.254	0.983	0	3	Formative index of school-facing parental involvement based on three binary indicators: parent-initiated teacher contact, teacher-initiated parent contact, and class parent committee membership. Possible scores range from 0 to 3, with higher scores indicating more forms of school-facing involvement
Parental Involvement (binary)	0.709	0.454	0	1	Binary indicator derived from the three school-facing involvement items: 0 = no reported school-facing involvement; 1 = at least one reported form of school-facing involvement
Instrumental Variable					
Mother as Primary Learning Supervisor	0.644	0.479	0	1	Whether mother is the main person supervising child’s learning at home 0 = No, 1 = Yes
Mediating Variable					
Public Praise	2.456	2.767	0	10	Number of times student is praised by teacher in front of the class in last month
Moderating Variable					
Emotional Sensitivity	5.596	1.753	1	5	Average of ten reverse-coded Likert items capturing students’ tendency to perceive, understand, and be affected by others’ emotions. Each item ranges from 1 to 5 after recoding, with higher values indicating greater emotional sensitivity.
Student-Level Control Variables					
Student Gender	0.541	0.498	0	1	0 = female, 1 = male
Student Age	11.408	1.059	9	12	Calculated by subtracting the student’s year of birth from the survey year
Previous Semester Academic Performance	4.323	0.779	1	5	Average of the student’s Chinese and mathematics grades from the previous semester, coded as: 1 = E, 2 = D, 3 = C, 4 = B, 5 = A
Class-Level Control Variables					
Homeroom Teacher Gender	0.136	0.343	0	1	0 = female, 1 = male
Homeroom Teacher’s Years of Teaching Experience	14.082	9.837	1	41	Calculated by subtracting the year the teacher began teaching from the survey year
Homeroom Teacher’s Professional Title	2.497	0.756	1	4	1 = No title, 2 = Level II, 3 = Level I, 4 = Senior title
Class Size	45.509	5.603	20	60	Number of students in the class
Family-Level Control Variables					
Parent Age	39.011	7.044	21	61	Calculated by subtracting the parent’s year of birth from the survey year.
Parents’ Highest Educational Attainment	5.096	1.677	1	8	Highest educational attainment of any household member, coded as: 1 = Illiterate, 2 = Primary school, 3 = Junior high school, 4 = General high school, 5 = Secondary technical/vocational school, 6 = Junior college, 7 = Bachelor’s degree, 8 = Master’s degree or above.
Parents’ Full-Time Employment	0.563	0.496	0	1	0 = No, 1 = Yes
Child’s Urban Hukou	0.538	0.499	0	1	0 = Agricultural hukou, 1 = Urban hukou
Child’s Local Hukou	0.666	0.472	0	1	0 = Non-local, 1 = Local
Annual Per Capita Household Income	3.577	1.349	1	6	1 = Less than 10,000 RMB, 2 = 10,000–20,000 RMB, 3 = 25,000–50,000 RMB, 4 = 50,000–100,000 RMB, 5 = 100,000–250,000 RMB, 6 = More than 250,000 RMB
Household Book Collection Size	3.582	1.864	1	8	1 = 10 books or fewer, 2 = 11–50 books, 3 = 51–100 books, 4 = 101–150 books, 5 = 151–200 books, 6 = 201–300 books, 7 = 301–400 books, 8 = More than 400 books.
Proportion of Child’s Books in Household Collection	0.822	0.193	0.13	1	Proportion of books in the household collection that are suitable for children’s reading
Parents’ Occupation Type	5.382	2.537	1	12	Parent’s current full-time occupation type: 1 = Self-employed, 2 = Entry-level laborer, 3 = Farmer/fisher/herder, 4 = General worker, 5 = Skilled worker, 6 = Business and service staff, 7 = General staff/clerical worker, 8 = Technical staff (e.g., accountant, nurse, software programmer), 9 = Professional (e.g., doctor, lawyer, primary/secondary school teacher), 10 = Senior professional (e.g., scientist, engineer, university teacher), 11 = Leader/manager in public institution or enterprise, 12 = Leader/official in government agency
Only Child	0.629	0.483	0	1	0 = No, 1 = Yes
Household Size	3.901	1.147	1	6	Total number of household members, with households of more than six members coded as 6.
Proportion of School-Age Children in Household	0.376	0.152	0.167	0.833	Proportion of school-age children (excluding kindergarten and preschool) in the total household population.

Note: N = 15,655 for all variables. Descriptive statistics are based on observed data. Regression analyses were conducted using complete cases after listwise deletion of observations with missing values on variables included in the corresponding model.

**Table 2:** Baseline OLS regression results.

Variable	(1) School Bullying	(2) School Bullying
Parental Involvement	0.147 *** (0.055)	0.222 *** (0.056)
Control Variables		√
Constant		15.996 *** (0.993)
95% CI	[0.112, 0.332]	
Observations	15,655	15,655
R^2^	0.008	0.055

Note. School-level cluster-robust standard errors are reported in parentheses. *** *p* < 0.01. The same applies below.

**Table 3:** Results of the instrumental-variable regressions.

Variable	(3) Parental Involvement	(4) School Bullying
Instrumental Variable	0.239 *** (0.017)	
Parental Involvement		1.818 *** (0.512)
Control Variables	√	√
Constant	0.501 *** (0.138)	17.357 *** (1.059)
First-stage coefficient 95% CI	[0.206, 0.272]	
Weak instrumental variable test (F value)	202.31 ***	
DWH endogeneity test (*p* value)	0.000	
Observations	15,655	
Partial R^2^	0.013	

Note: School-level cluster-robust standard errors are reported in parentheses. Weak-instrument diagnostics are based on the first-stage specification with the same covariates. The model is exactly identified; therefore, an overidentification test is not applicable. Controlled Variables include Student-Level, Class-Level, and Family-Level Control Variables, and the same is shown below. *** *p* < 0.01.

**Table 4:** Regression Results of the Mediation and Moderation Analyses.

Variable	(5) Publicly Praised	(6) School Bullying	(7) School Bullying	(8) School Bullying	(9) School Bullying
	Mediating Association			Moderating Association	
Parental Involvement	0.141 *** (0.023)		0.208 *** (0.056)	0.213 *** (0.057)	0.211 *** (0.058)
Publicly Praised		0.105 *** (0.020)	0.101 *** (0.020)		
Emotional Sensitivity				0.356 *** (0.095)	0.351 *** (0.096)
Parental Involvement × Emotional Sensitivity					0.074 *** (0.029)
Control Variables	√	√	√	√	√
Constant	−0.498 (0.404)	16.187 *** (0.991)	16.047 *** (0.992)	14.974 *** (1.102)	15.608 *** (1.049)
*Sobel Z*	3.431 ***				
*Bootstrap CI*	[0.006, 0.023]				
Observations	15,655	15,655	15,655	15,655	15,655
R^2^	0.035	0.056	0.057	0.056	0.057

Note: School-level cluster-robust standard errors are reported in parentheses. *** *p* < 0.01.

**Table 5:** Heterogeneity Results by Only-Child Status and Urban Hukou.

Variable	(10) School Bullying	(11) School Bullying
Parental Involvement (binary)	−0.363 (0.231)	−0.210 (0.211)
Only Child	−0.412 ** (0.204)	
Parental Involvement (binary) × Only Child	0.618 ** (0.288)	
Urban Hukou		−0.384 * (0.216)
Parental Involvement (binary) × Urban Hukou		0.518 * (0.284)
Control Variables	√	√
Constant	16.139 *** (0.997)	16.173 *** (0.994)
Observations	15,655	15,655
R^2^	0.056	0.056

Note: School-level cluster-robust standard errors are reported in parentheses. The reference category for parental involvement is no involvement. Separate interaction models were also estimated for local versus non-local hukou status, annual per capita household income, and household book collection size. The joint Wald tests of the interaction terms were not statistically significant for these dimensions. For parsimony, Table 5 presents only-child status and urban hukou, the two dimensions showing the strongest exploratory heterogeneity patterns. These heterogeneity analyses are exploratory, and *p*-values are not adjusted for multiple testing. *** *p* < 0.01, ** *p* < 0.05, * *p* < 0.1.

## Data Availability

The data supporting the findings of this study are not publicly available because they contain information on minors and are subject to ethical and privacy restrictions. The de-identified analytic variables used in the analyses and the replication code may be made available from the corresponding author upon reasonable request, subject to institutional approval and a data-use agreement.

## Supplementary Materials

The following supporting information can be downloaded at: https://www.mdpi.com/article/10.3390/bs16081267/s1, The following supporting information is available in the supplementary file: Table S1: Robustness Checks Using the Binary Measure of Parental Involvement; Table S2: Items and Coding of the Main Measures; Table S3: Distribution and Correlations of the Components of Parental Involvement; Table S4: Separate Analyses of the Components of Parental Involvement; Table S5: Sensitivity Analysis Using the Dichotomized Emotional-Sensitivity Measure; Table S6: Decomposition of Total, Direct, and Indirect Associations in the Mediation Analysis; Table S7: Robustness Checks Using Negative Binomial Regression; Table S8: Marginal Effects and Predicted Values for Exploratory Heterogeneity Analyses.
