# Supplementary material for: Parental Involvement, Teacher Public Praise, and Bullying Victimization Among Primary School Students: Evidence from China
Source: Behav Sci (Basel). 2026 Jul 23;16(8):1267. doi: 10.3390/bs16081267 (PMC13509898; doi:10.3390/bs16081267)
Supplement: Supplementary file 1 [file behavsci-16-01267-s001.zip › behavsci-4386569-supplementary.pdf]

Supplementary Table S1. Robustness Checks Using the Binary Measure of Parental Involvement.

Variable	（12）<br>**School bullying**	（13）<br>**School bullying**
Parental Involvement (binary)	0.308*** (0.094)	0.329*** (0.120)
Student Gender		1.196*** (0.105)
Student Age		-0.146*** (0.052)
Previous Semester Academic Performance		-1.208*** (0.075)
Parents' Age		0.007 (0.008)
Parents' Highest Educational Attainment		0.017 (0.040)
Parents' Full-Time Employment		-0.144 (0.109)
Child's Urban Hukou		0.052 (0.134)
Child's Local Hukou		-0.189 (0.136)
Annual Per Capita Household Income		-0.004 (0.043)
Household Book Collection Size		-0.186*** (0.032)
Proportion of Child's Books in Household Collection		-0.996*** (0.283)
Parents' Occupation Type		-0.020 (0.022)
Only Child		-0.162 (0.128)
Household Size		-0.074 (0.062)
Proportion of School-Age Children in Household		-0.318 (0.442)
Homeroom Teacher Gender		-0.951*** (0.164)
Homeroom Teacher's Years of Teaching Experience		-0.013 (0.008)
Homeroom Teacher's Title		-0.021 (0.101)
Class Size		0.003

		(0.009)
Constant		16.011***
		(0.994)
Observations	15655	15655
R^2	0.008	0.054

Note: School-level cluster-robust standard errors are reported in parentheses.

Supplementary Table S2. Items and Coding of the Main Measures.

**Construct**	**Item**	**Original item**	**Response options and recoding**
School bullying	stu_86_a	Classmates teased me or verbally abused me.	3 = at least once a week; 2 = once or twice a month; 1 = a few times a year; 0 = never or almost never
	stu_86_b	Other classmates did not let me join when they played together.	3 = at least once a week; 2 = once or twice a month; 1 = a few times a year; 0 = never or almost never
	stu_86_c	Classmates spread rumors about me.	3 = at least once a week; 2 = once or twice a month; 1 = a few times a year; 0 = never or almost never
	stu_86_d	Classmates stole my belongings.	3 = at least once a week; 2 = once or twice a month; 1 = a few times a year; 0 = never or almost never
	stu_86_e	Classmates hurt me, such as by kicking, hitting, or pushing me.	3 = at least once a week; 2 = once or twice a month; 1 = a few times a year; 0 = never or almost never
	stu_86_f	Classmates forced	3 = at least once a

		me to do things I did not want to do.	week; 2 = once or twice a month; 1 = a few times a year; 0 = never or almost never
	stu_86_g	Classmates spread embarrassing information about me.	3 = at least once a week; 2 = once or twice a month; 1 = a few times a year; 0 = never or almost never
	stu_86_h	Classmates threatened me.	3 = at least once a week; 2 = once or twice a month; 1 = a few times a year; 0 = never or almost never
Parental involvement	parent_74	In the past month, did you or other family members actively ask the teacher about the student's situation at school?	1 = Yes; 0 = No
	parent_75	In the past month, did the school contact you or other family members individually about the student's situation at school?	1 = Yes; 0 = No
	parent_76	Are you or other family members members of the class parent committee?	1 = Yes; 0 = No or no parent committee
Emotional sensitivity	stu_85_b	After spending time with a friend who is feeling down, I usually feel sad.	5 = Strongly agree; 4 = Somewhat agree; 3 = Uncertain; 2 = Somewhat disagree; 1 = Strongly disagree

	stu_85_d	I feel afraid when I see some scenes in horror movies.	5 = Strongly agree; 4 = Somewhat agree; 3 = Uncertain; 2 = Somewhat disagree; 1 = Strongly disagree
	stu_85_e	I am easily affected by other people's emotions.	5 = Strongly agree; 4 = Somewhat agree; 3 = Uncertain; 2 = Somewhat disagree; 1 = Strongly disagree
	stu_85_i	When someone is feeling down, I can usually understand how they feel.	5 = Strongly agree; 4 = Somewhat agree; 3 = Uncertain; 2 = Somewhat disagree; 1 = Strongly disagree
	stu_85_j	I can usually notice when my friend feels afraid.	5 = Strongly agree; 4 = Somewhat agree; 3 = Uncertain; 2 = Somewhat disagree; 1 = Strongly disagree
	stu_85_k	I often feel sad when I see sad scenes on television or in movies.	5 = Strongly agree; 4 = Somewhat agree; 3 = Uncertain; 2 = Somewhat disagree; 1 = Strongly disagree
	stu_85_l	I can often sense people's emotions before they tell me how they feel.	5 = Strongly agree; 4 = Somewhat agree; 3 = Uncertain; 2 = Somewhat disagree; 1 = Strongly disagree
	stu_85_o	When my friend feels afraid, I often	5 = Strongly agree; 4 = Somewhat

		feel afraid as well.	agree; 3 = Uncertain; 2 = Somewhat disagree; 1 = Strongly disagree
	stu_85_p	I can usually notice when my friend is angry.	5 = Strongly agree; 4 = Somewhat agree; 3 = Uncertain; 2 = Somewhat disagree; 1 = Strongly disagree
	stu_85_q	I am often affected by my friends' emotions.	5 = Strongly agree; 4 = Somewhat agree; 3 = Uncertain; 2 = Somewhat disagree; 1 = Strongly disagree

Note: For the school bullying measure, all eight items were reverse-coded from 0 to 3 and summed to create an index ranging from 0 to 24, with higher scores indicating more frequent bullying-related victimization. For parental involvement, the three binary indicators were summed to create a continuous involvement-intensity index ranging from 0 to 3. A binary parental-involvement indicator was also constructed, with 0 indicating no reported school-facing involvement and 1 indicating at least one reported form of involvement. For emotional sensitivity, the ten items were reverse-coded so that higher values indicated greater emotional sensitivity. The recoded items were averaged to create a continuous score ranging from 1 to 5. This continuous score is used in the main moderation analysis. For the supplementary sensitivity analysis only, we also constructed a dichotomized version, with students above the sample mean coded as 1 and those at or below the sample mean coded as 0. This dichotomized measure is not based on a validated clinical or psychometric threshold and is used only to assess sensitivity to the earlier operationalization.

Supplementary Table S3. Distribution and Correlations of the Components.

Panel A. Distribution of the three components.

**Component**	**Description**	**Mean**	**SD**	**Percentage = 1**
Parent-initiated teacher contact	Parents or family members actively asked teachers about the student's situation at school during the past month	0.560	0.496	56.0%
Teacher-initiated	The school contacted parents	0.490	0.500	49.0%

parent contact	or family members individually about the student's situation at school during the past month			
Class parent committee membership	Parents or family members served as members of the class parent committee	0.204	0.403	20.4%

Panel B. Pairwise correlations among the three components.

Component	1	2	3
Parent-initiated teacher contact	1.000		
Teacher-initiated parent contact	0.305	1.000	
Class parent committee membership	0.154	0.099	1.000

Supplementary Table S4. Separate Analyses of the Components of Parental Involvement.

Variable	Model S4-1	Model S4-2	Model S4-3	Model S4-4
Parent-initiated teacher contact	0.210* (0.111)			0.041 (0.118)
Teacher-initiated parent contact		0.467*** (0.127)		0.431*** (0.141)
Class parent committee membership			0.289** (0.131)	0.211* (0.121)
Control variables	√	√	√	√
Observations	15,655	15,655	15,655	15,655
R^2	0.055	0.057	0.056	0.058

Note: School-level cluster-robust standard errors are reported in parentheses.

Supplementary Table S5. Sensitivity Analysis Using the Dichotomized Emotional-Sensitivity Measure.

Variable	Model S5-1 School Bullying	Model S5-2 School Bullying
Parental Involvement	0.112** (0.050)	0.198** (0.078)
Emotional Sensitivity (binary)	0.846** (0.351)	1.121** (0.507)
Parental Involvement × Emotional Sensitivity (binary)		0.128** (0.055)
Control variables	√	√
Observations	15,655	15,655
R^2	0.058	0.059

Note: This table reports sensitivity analyses using the dichotomized emotional-

sensitivity measure. The main analysis treats emotional sensitivity as a continuous measure. School-level cluster-robust standard errors are reported in parentheses.

Supplementary Table S6. Decomposition of Total, Direct, and Indirect Associations in the Mediation Analysis.

**Effect**	**Estimate**	**Bootstrap SE**	**95% Bootstrap CI**	**p-value**
Total effect	0.222	0.058	[0.109, 0.336]	< 0.001
Direct effect	0.208	0.056	[0.099, 0.317]	< 0.001
Indirect effect through public praise	0.014	0.004	[0.006, 0.023]	0.001
Proportion mediated	6.4%			

Note: The indirect association was estimated using 5,000 bootstrap resamples. The Sobel test was retained only as a supplementary check and was not used as the primary evidence for mediation. The mediation analysis should be interpreted as exploratory statistical mediation rather than causal mediation because the data are cross-sectional and the mediation model does not fully incorporate the instrumental-variable identification strategy.

Supplementary Table S7. Robustness Checks Using Negative Binomial Regression.

Variable	Model S7-1 School Bullying	Model S7-2 School Bullying	Model S7-3 School Bullying
Parental Involvement	0.034** (0.015)	0.032** (0.015)	0.033** (0.015)
Public Praise		0.016*** (0.004)	
Emotional Sensitivity			0.054*** (0.018)
Parental Involvement × Emotional Sensitivity			0.011** (0.005)
Control variables	√	√	√
Observations	15,655	15,655	15,655
Pseudo R^2	0.020	0.022	0.023
Alpha	0.46***	0.45***	0.45***

Note: Negative binomial models are used as robustness checks for the bounded and moderately right-skewed school bullying score. Coefficients are reported in log-count units. School-level cluster-robust standard errors are reported in parentheses. *** $p < 0.01$, ** $p < 0.05$, * $p < 0.10$.

Supplementary Table S8. Marginal Effects and Predicted Values for Exploratory Heterogeneity Analyses.

Panel A. Marginal effects of parental involvement by subgroup.

Model	Subgroup	Marginal effect of parental involvement	SE	95% CI	p-value
Only-child status	Students with siblings	-0.363	0.231	[-0.829, 0.103]	0.123
Only-child status	Only children	0.255	0.119	[0.015, 0.495]	0.038
Urban hukou	Rural hukou students	-0.210	0.211	[-0.636, 0.216]	0.326
Urban hukou	Urban hukou students	0.308	0.152	[0.002, 0.614]	0.049

Panel B. Average adjusted predicted values.

Model	Subgroup	No parental involvement	Any parental involvement	Difference
Only-child status	Students with siblings	6.650	6.287	-0.363
Only-child status	Only children	6.238	6.493	0.255
Urban hukou	Rural hukou students	6.700	6.490	-0.210
Urban hukou	Urban hukou students	6.316	6.624	0.308

Note: Marginal effects and predicted values are based on the models reported in Table 5. Predicted values are average adjusted predictions averaged over the observed covariate distribution. School-level cluster-robust standard errors are used. These heterogeneity analyses are exploratory, and p-values are not adjusted for multiple testing.
